# Impact of Regulated and Non-Regulated Food-Associated Mycotoxins on the Viability and Proliferation of Enteric Glial Cells

**DOI:** 10.3390/toxins17120587

**Published:** 2025-12-08

**Authors:** Michał Dąbrowski, Hamza Olleik, Attilio Di Maio, Amine Kadri, Valérie Camps, Josette Perrier, El Hassan Ajandouz, Philippe Pinton, Regiane R. Santos, Isabelle P. Oswald, Łukasz Zielonka, Marc Maresca

**Affiliations:** 1 Department of Veterinary Prevention and Feed Hygiene, Faculty of Veterinary Medicine, University of Warmia and Mazury in Olsztyn, Oczapowski Str. 13/29, 10-718 Olsztyn, Poland; michal.dabrowski@uwm.edu.pl (M.D.); lukaszz@uwm.edu.pl (Ł.Z.); 2 Aix Marseille Univ., CNRS, Centrale Marseille, iSm2, 13013 Marseille, France; hamza.olleik@live.com (H.O.); attilio742@gmail.com (A.D.M.); keops1986@icloud.com (A.K.); camps.valerie@orange.fr (V.C.); josette.perrier@univ-amu.fr (J.P.); el-hassan.ajandouz@univ-amu.fr (E.H.A.); 3 Toxalim, Research Center in Food Toxicology, Université de Toulouse, INRAE, ENVT, INP-PURPAN, UPS, 31027 Toulouse, France; philippe.pinton@inrae.fr (P.P.); isabelle.oswald@inrae.fr (I.P.O.); 4 Schothorst Feed Research, P.O. Box 533, 8200 AM Lelystad, The Netherlands; rsantos@schothorst.nl

**Keywords:** mycotoxins, emerging mycotoxins, non-regulated mycotoxins, enteric nervous system, enteric glial cells, cyclohexadepsipeptide

## Abstract

(1) Background: Humans and animals are exposed daily to numerous food-associated noxious molecules, including fungal toxins or mycotoxins. Effects of mycotoxins on the intestinal epithelial cells (IECs) are well characterized. However, their impact on the enteric nervous system (ENS), particularly on enteric glial cells (EGCs), has not been evaluated. (2) Methods: In the present work, the impact of major mycotoxins (eighteen mycotoxins in total, both regulated and non-regulated (including emerging ones) mycotoxins) on EGCs was evaluated in vitro in terms of antiproliferative and cytotoxic effects using rat EGCs as a model. Inhibitory concentrations on cell division and cell viability were determined using the resazurin assay, and biochemical analysis was performed to identify the mechanism(s) of action involved. (3) Results: Of the eighteen mycotoxins tested, twelve were found to be toxic; apicidin, deoxynivalenol, and cyclohexadepsipeptide mycotoxins (enniatins and beauvericin) were the most toxic, with active concentrations as low as 0.19 ± 0.07 µM for deoxynivalenol. Mechanistic studies revealed that toxicity occurs through the induction of oxidative stress, alteration of the membrane integrity, and/or induction of apoptosis. (4) Conclusions: As far as we know, the data presented here show for the first time that EGCs are targets of foodborne mycotoxins, even at low concentrations potentially achieved in cases of ingesting contaminated food.

## 1. Introduction

**Figure 1:**
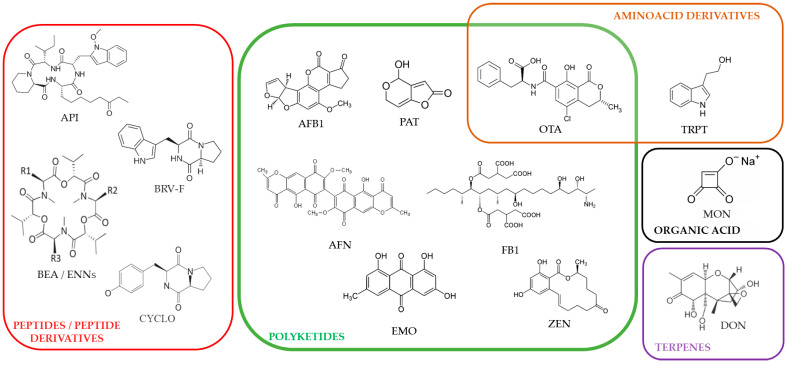
Chemical structures of the regulated and non-regulated food-associated mycotoxins tested in this study: aflatoxin B1 (AFB1), apicidin (API), 56 aurofusarin (AFN), beauvericin (BEA), brevianamide-F (BRV-F), cyclo-(L-Pro-L-Tyr) (CYCLO), deoxynivalenol (DON), emodin (EMO), enniatins (ENNs), fumonisin B1 (FB1), moniliformin (MON), ochratoxin A (OTA), patulin (PAT), tryptophol (TRPT), and zearalenone (ZEN).

Mycotoxins, both regulated and non-regulated (including “emerging mycotoxins”), are produced by various fungi infecting plants and crops [1,2,3,4]. As a consequence, they frequently contaminate food and feed, causing daily exposure to humans and animals through ingestion [5,6]. The intestine is the first and most exposed tissue during food-associated mycotoxicosis. The effects of mycotoxins on the viability and functions of intestinal epithelial cells (IECs) have been extensively documented [7,8,9,10,11,12]. Similarly, although less studied, the impact of mycotoxins on the viability and functions of brain cells has been described [13,14,15,16,17]. The enteric nervous system (ENS) corresponds to neurons and glial cells located within the intestinal wall, and, similarly to IECs, cells of the ENS are potentially exposed to very high doses of mycotoxins. Surprisingly, only a few studies have investigated the impact of those toxins on cells of this system, including enteric glial cells (EGCs) [18,19,20,21]. EGCs are major players in the formation, homeostasis, and function of the ENS and are thus crucial for overall gut physiology. Notably, crosstalk between EGCs and IECs helps establish and maintain the intestinal barrier and immune function [22,23,24,25,26,27]. To date, only one study has described the impact of the mycotoxin deoxynivalenol (DON) on EGCs [28]. In this pioneer study, Rissato et al. were the first to show that DON alters EGCs in vivo in rats. Based on that ascertainment, in the present study, we evaluated the effect of eighteen major mycotoxins on EGCs in vitro using rat EGCs as a model. Mycotoxins tested and shown in Figure 1 correspond to both regulated and non-regulated ones. They were selected from a global mycotoxin survey, where more than 1100 feed samples from 44 countries were analyzed using a multi-analyte LC-MS/MS method. They were chosen based on their occurrence, their toxic effect on animals and/or humans, and the fact that they are commercially available [5].

The six regulated toxins tested, based on their high prevalence and levels in human and animal foods [5], were aflatoxin B1 (AFB1), deoxynivalenol (DON), fumonisin B1 (FB1), ochratoxin A (OTA), patulin (PAT), and zearalenone (ZEN). The eleven non-regulated mycotoxins tested, also due to their high prevalence and levels in food/feed [5], were apicidin (API), aurofusarin (AFN), beauvericin (BEA), brevianamide-F (BRV-F), cyclo-(L-Pro-L-Tyr) (CYCLO), emodin (EMO), enniatins (ENNs) (A, A1, B, B1), moniliformin (MON), and tryptophol (TRPT). Data shows that twelve out of the eighteen mycotoxins tested can alter the proliferation and/or survival of EGCs through oxidative stress, membrane alteration, and/or apoptosis.

## 2. Results

### 2.1. Regulated and Non-Regulated Mycotoxins Alter EGC Proliferation

**Figure 2:**
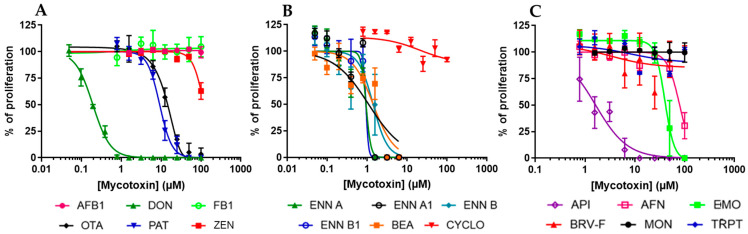
Effect of mycotoxins on the proliferation of EGCs. Dividing EGCs were exposed to increasing concentrations of regulated (**A**) or non-regulated mycotoxins (**B**,**C**) for 48 h before evaluation of the cell proliferation using the resazurin assay. Results are expressed as a percentage of proliferation (means ± SD), with the negative controls (vehicle alone) giving 100% proliferation (*n* = 3).

To evaluate the antiproliferative effect of mycotoxins, dividing EGCs were exposed to increasing concentrations of mycotoxins for 48 h. At the end of the exposure, cell density was measured using the resazurin assay. The inhibitory concentration 50% values (IC_50_), i.e., the concentration causing a reduction of 50% of the cell proliferation, were graphically determined using GraphPad Prism 10 software (Figure 2 and Table 1). Data showed that of the six regulated mycotoxins tested, only three, i.e., DON, OTA, and PAT, were able to inhibit the division of EGCs with IC_50_ values of 0.19 ± 0.07, 13.79 ± 1.13, and 9.19 ± 0.96 µM, respectively. The three other regulated mycotoxins tested (i.e., AFB1, FB1, and ZEN) were found to be less toxic or not toxic, with IC_50_ higher than 100 µM (Figure 2A).

Regarding the non-regulated mycotoxins, the data showed that ENNs and BEA were the most active, with IC_50_ values ranging from 0.92 ± 1.07 to 1.41 ± 0.20 µM (Figure 2B). AFN, API, and EMO were also found to inhibit the proliferation of EGCs with IC_50_ values of 79.51 ± 3.68, 1.63 ± 0.21, and 40.01 ± 2.70 µM, respectively. On the contrary, CYCLO, BRV-F, MON, and TRPT did not affect cell division for concentrations up to 100 µM (Figure 2C).

### 2.2. Regulated and Non-Regulated Mycotoxins Affect EGC Viability

**Figure 3:**
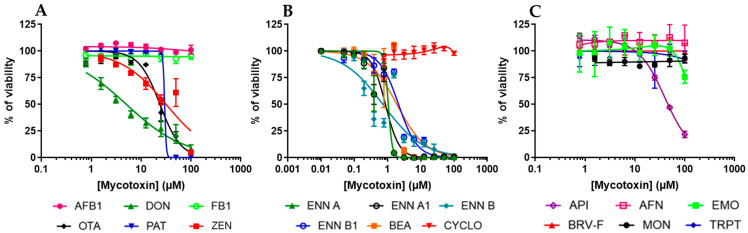
Effect of mycotoxins on the viability of EGCs. Confluent EGCs were exposed to increasing concentrations of regulated (**A**) or non-regulated mycotoxins (**B**,**C**) for 48 h before evaluation of the living cell viability using resazurin. Results are expressed as a percentage of viability (means ± SD), with the negative controls (vehicle alone) giving 100% viability (*n* = 3).

The effect of mycotoxins on EGC viability was then measured by exposing confluent/non-dividing EGCs to increasing concentrations of toxins for 48 h, allowing the determination of their cytotoxic concentration 50% values (CC_50_) (i.e., the concentrations causing a 50% decrease in cell viability) (Figure 3 and Table 2). Among regulated mycotoxins (Figure 3A), DON, OTA, PAT, and ZEN decreased cell viability, giving CC_50_ of 5.06 ± 0.48, 23.88 ± 1.36, 38.02 ± 11.37, and 31.75 ± 4.94 µM, respectively. Regarding the non-regulated mycotoxins (Figure 3B,C), only API (CC_50_ value of 59.59 ± 10.27 µM) and the cyclohexadepsipeptides (ENNs and BEA) inhibited EGC viability with CC_50_ values ranging from 0.72 ± 0.16 to 2.14 ± 0.17 µM. The other regulated or non-regulated mycotoxins tested (i.e., AFB1, AFN, BRV-F, CYCLO, EMO, FB1, MON, and TRPT) did not cause cytotoxicity at concentrations up to 100 µM.

### 2.3. Oxidative Stress, Membrane Alteration, and/or Apoptosis Induction Are Involved in the Toxic Effect of Regulated and Non-Regulated Mycotoxins on EGCs

Regulated and non-regulated mycotoxins causing toxicity to EGCs were further studied through mechanistic approaches, including measurement of cellular oxidative stress, membrane permeabilization, and induction of apoptosis. All assays were performed after exposure of EGC to concentrations of mycotoxins close to their CC_50_ on EGC viability and at 100 µM of toxins.

**Figure 4:**
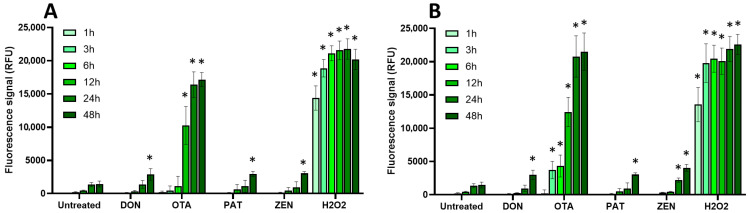
Effect of regulated mycotoxins on ROS production of EGCs. Confluent EGCs were loaded with DCFDA probe (25 µM) to detect intracellular ROS. Loaded cells were then exposed to regulated mycotoxins shown to be toxic to EGCs at concentrations close to their CC_50_ on cell viability (i.e., 5 µM of DON, 25 µM of OTA, 40 µM of PAT, or 30 µM of ZEN) (**A**) or at 100 µM (**B**). H_2_O_2_ at 500 µM final concentration was used as a positive control for the induction of intracellular ROS production. After various exposure times, fluorescence was measured (Ex 485 nm/Em 535 nm). Results are expressed as means ± SD (*n* = 3). * *p* < 0.05 compared with control cells without mycotoxin at the same time.

**Figure 5:**
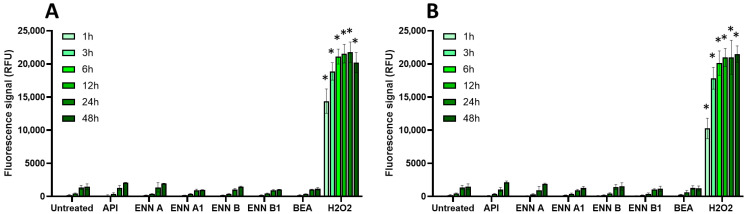
Effect of non-regulated mycotoxins on ROS production of EGCs. Confluent EGCs were loaded with DCFDA probe (25 µM) to detect intracellular ROS. Loaded cells were then exposed to non-regulated mycotoxins shown to be toxic to EGCs at concentrations close to their CC_50_ on cell viability (i.e., 60 µM of API, 1 µM of ENN A, A1 or B, 2 µM of ENN B1 or BEA) (**A**) or at 100 µM (**B**). H_2_O_2_ at 500 µM final concentration was used as a positive control for the induction of intracellular ROS production. After various exposure times, fluorescence was measured (Ex 485 nm/Em 535 nm). Results are expressed as means ± SD (*n* = 3). * *p* < 0.05 compared with control cells without mycotoxin at the same time.

Cellular oxidative stress was measured using the DCFDA assay as explained in the Materials and Methods Section (Figure 4 and Figure 5).

Among the regulated mycotoxins toxic to EGCs, OTA, both at 25 µM (a concentration close to its CC_50_ on EGC viability) and at 100 µM, caused the most significant increase in the production of intracellular radical oxygen species (ROS), with signals comparable to those caused by H_2_O_2_ used as a positive control (Figure 4). At 25 µM, the effect of OTA on ROS production started after 12 h exposure and was maximal at 24–48 h after exposure, whereas at 100 µM of OTA, significant ROS production was observed as soon as 3 h after exposure. The other regulated mycotoxins found to be toxic to EGCss (i.e., DON, PAT, and ZEN) also caused ROS production but with a lower effect and only after 48 h of exposure (except for ZEN at 100 µM, where a significant increase was observed after 24 h of exposure).

Regarding the non-regulated mycotoxins that are toxic to EGCs, none of the tested compounds, either at their CC_50_ values or at 100 μM, caused a significant increase in intracellular ROS production, even after 48 h of exposure. This suggests that oxidative stress plays no role in their toxicity to EGCs or, at least, that it cannot be detected under the specific conditions used here (Figure 5).

**Figure 6:**
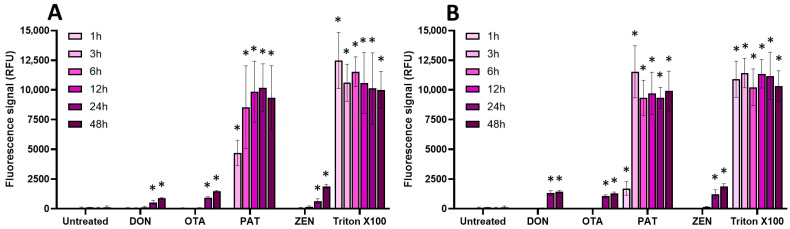
Effect of regulated mycotoxins on the membrane integrity of EGCs. Confluent EGCs were exposed to regulated mycotoxins shown to be toxic to EGCs at concentrations close to their CC_50_ on cell viability (i.e., 5 µM of DON, 25 µM of OTA, 40 µM of PAT, or 30 µM of ZEN) (**A**) or at 100 µM (**B**). Membrane integrity was measured using propidium iodide, with Triton X-100 at 0.1% (*v*:*v*) final concentration being used as a positive control for membrane permeabilization. After various exposure times, fluorescence was measured (Ex 530 nm/Em 590 nm) to monitor the PI signal. Results are expressed as means ± SD (*n* = 3). * *p* < 0.05 compared with control cells without mycotoxin at the same time.

**Figure 7:**
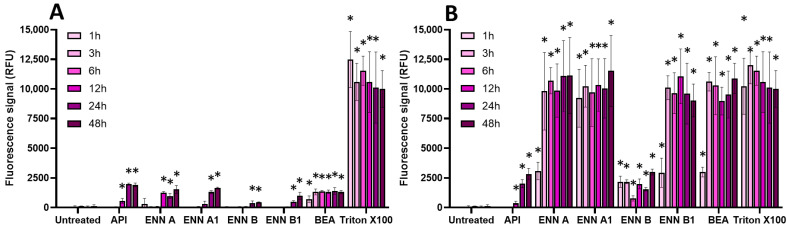
Effect of non-regulated mycotoxins on the membrane integrity of EGCs. Confluent EGCs were exposed to non-regulated mycotoxins shown to be toxic to EGCs at concentrations close to their CC_50_ on cell viability (i.e., 60 µM of API, 1 µM of ENN A, A1 or B, 2 µM of ENN B1 or BEA) (**A**) or at 100 µM (**B**) in the presence of propidium iodide. Membrane integrity was measured using propidium iodide, with Triton X-100 at 0.1% (*v*:*v*) final concentration being used as a positive control for membrane permeabilization. After various exposure times, fluorescence was measured (Ex 530 nm/Em 590 nm) to monitor the PI signal. Results are expressed as means ± SD (*n* = 3). * *p* < 0.05 compared with control cells without mycotoxin at the same time.

Since some mycotoxins, such as ENN and BEA, have been reported to compromise membrane integrity in different cell types through their ionophore mode of action [29], the effect of mycotoxins on the membrane integrity of EGCs was then investigated using the propidium iodide (PI) assay (Figure 6 and Figure 7).

Regarding regulated mycotoxins (Figure 6), PAT (at its CC_50_ and at 100 µM) was the only one to cause a significant membrane permeabilization (similar to the positive control Triton X-100), starting after 1 h of incubation and maximal at 3–6 h. This is in agreement with previous data showing that PAT compromises membrane integrity [30]. DON, OTA, and ZEN were much less potent at causing membrane damage, with only a weak but still significant effect after 24 h of incubation.

Concerning non-regulated mycotoxins (Figure 7), as expected due to their ionophore mode of action [29], at 100 µM, ENN A, ENN A1, ENN B1, and BEA were the most active in terms of membrane permeabilization, starting after 1 h of exposure and with maximal effect after 3 h of incubation, giving a similar signal compared to Triton X-100. At the same concentration, API and ENN B caused less membrane damage. At their CC_50_, all non-regulated mycotoxins were still able to permeabilize the membrane of EGCs, albeit with a lower effect; significant effects were observed after 12–48 h of incubation, except for BEA, which acted faster (starting after 1 h of contact).

**Figure 8:**
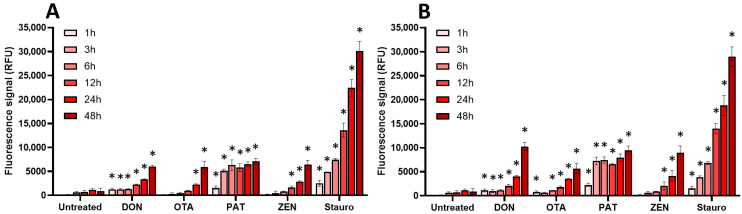
Effect of regulated mycotoxins on caspase-3/7 activation of EGCs. Confluent EGCs were exposed to regulated mycotoxins shown to be toxic to EGCs at concentrations close to their CC_50_ on cell viability (i.e., 5 µM of DON, 25 µM of OTA, 40 µM of PAT, or 30 µM of ZEN) (**A**) or at 100 µM (**B**). Caspase-3/7 activation was measured using the CellEvent kit, with staurosporine (Stauro) at 10 µM final concentration being used as a positive control for caspase activation. After various exposure times, fluorescence was measured (Ex 500 nm/Em 530 nm) to monitor the PI signal. Results are expressed as means ± SD (*n* = 3). * *p* < 0.05 compared with control cells without mycotoxin at the same time.

**Figure 9:**
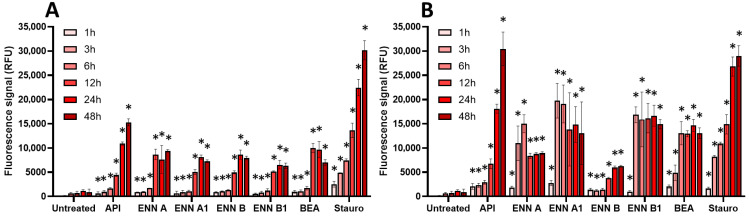
Effect of non-regulated mycotoxins on caspase-3/7 activation of EGCs. Confluent EGCs were exposed to non-regulated mycotoxins shown to be toxic to EGCs at concentrations close to their CC_50_ on cell viability (i.e., 60 µM of API, 1 µM of ENN A, A1 or B, 2 µM of ENN B1 or BEA) (**A**) or at 100 µM (**B**). Caspase-3/7 activation was measured using the CellEvent kit, with staurosporine (Stauro) at 10 µM final concentration being used as a positive control for caspase activation. After various exposure times, fluorescence was measured (Ex 500 nm/Em 530 nm) to monitor the PI signal. Results are expressed as means ± SD (*n* = 3). * *p* < 0.05 compared with control cells without mycotoxin at the same time.

Finally, the effect of regulated and non-regulated mycotoxins on the induction of apoptosis in EGCs was measured using the caspase-3/7 assay (Figure 8 and Figure 9).

For regulated mycotoxins (Figure 8), DON, OTA, and ZEN (at their CC_50_ and at 100 µM) caused a time-dependent activation of caspase-3/7, with a maximal effect being observed after 48 h of exposure. PAT acted the fastest with a maximal effect after 3–6 h of incubation.

Among non-regulated mycotoxins (Figure 9), API caused the highest activation of caspase-3/7, with a similar signal compared to the positive control staurosporine. The induction of caspase-3/7 by API started at 1–3 h but required 48 h to reach maximal effect. ENNs and BEA also caused activation of caspase-3/7, the effect being significant after 6–12 h of incubation when EGCs were treated at their IC_50_. At 100 µM, induction was stronger in terms of signal but also faster, with maximal effect after 3–6 h of exposure, except for ENN B, which had a maximal effect after 24 h of exposure.

## 3. Discussion

In the present study, the toxicity of eighteen regulated and non-regulated/emerging mycotoxins on enteric glial cells was evaluated using rat EGCs as a model. These mycotoxins were selected on the basis of their occurrence in food and feed and their known or suspected toxicity to humans and/or animals [5,6]. As regulated mycotoxins, we evaluated the effect of AFB1, DON, FB1, OTA, PAT, and ZEN. For non-regulated mycotoxins, API, AFN, BEA, BRV-F, CYCLO, EMO, ENNs (ENN A, A1, B, B1), MON, and TRPT were tested.

Concerning their ability to inhibit EGC proliferation, among the eighteen mycotoxins evaluated, twelve toxins were able to inhibit the division of normal rat EGCs, i.e., DON, OTA, PAT, and ZEN for the regulated ones and API, AFN, BEA, EMO, and ENNs (ENN A, A1, B, and B1) for the non-regulated ones. An antiproliferative effect was observed at doses as low as 0.19 µM with the following order of IC_50_: DON < ENN A, A1, B1 < API < ENN B = BEA < PAT < OTA < EMO < AFN < ZEN (Table 1).

When testing the cytotoxicity of the toxins to EGCs, ten mycotoxins decreased the viability of EGCs, i.e., DON, OTA, PAT, and ZEN for the regulated ones and API, BEA, and ENNs (ENN A, A1, B, and B1) for the non-regulated ones. Cytotoxic effect was observed at doses ranging from 0.4 to 59.59 µM with the following order of CC_50_: ENN B < ENN A1 < ENN A < BEA < ENN B1 < DON < OTA < PAT < API (Table 1). AFB1, BRV-F, CYCLO, FB1, TRPT, and MON were all found not toxic to rat EGCs, at least for doses up to 100 µM and after 48 h of treatment.

Interestingly, results show that the sensitivity of rat EGCs to mycotoxins depends on their status, i.e., proliferating/dividing or confluent/non-dividing cells. OTA, BEA, and ENNs (ENN A, A1, and B1) gave very similar toxic concentrations on dividing and non-dividing cells, with a 0.77- to 1.36-fold difference between dividing (IC_50_) and non-dividing (CC_50_) EGCs. In contrast, API was more toxic to dividing than non-dividing cells with IC_50_ and CC_50_ of 1.25 and 59.59 µM, respectively (47.6-fold difference). AFN, EMO, DON, and PAT showed the same tendency, with those toxins being more toxic to dividing cells than to non-dividing ones (with a 3.5- and 26.6-fold difference for PAT and DON, respectively). Surprisingly, ENN B and ZEN were found to be more toxic for non-dividing cells than for dividing ones (1.94- and 3.71-fold difference, respectively). The reason for such differences could be related to differences in mycotoxin entry and/or detoxification efficiency. It could also be due to differences in the modulation of the expression of their molecular target(s) in dividing versus non-dividing EGCs. Future work will be necessary to investigate this point, as well as to identify the mechanism(s) by which mycotoxins cause toxic effects on EGCs.

In terms of mechanism(s) of toxicity, mechanistic approaches demonstrated that the toxic effect of regulated and non-regulated mycotoxins on EGCs relies on membrane damage, ROS production, and/or induction of caspase-3/7 and apoptosis. In terms of ROS production, OTA was more potent at causing oxidative stress to EGCs, a similar effect being described in other cell types [30]. Other regulated and non-regulated mycotoxins caused limited or no production of ROS, suggesting that they act through other mechanism(s). Regarding membrane permeabilization, PAT was the only regulated mycotoxin able to cause rapid (after 3 h) and significant membrane damage at its CC_50_ and at 100 µM. Other regulated mycotoxins caused weaker permeabilization, mainly observed at 100 µM and after 24–48 h of incubation. Among the non-regulated mycotoxins tested, and according to previous works [29,31], ENNs (except ENN B) and BEA caused significant membrane damage in EGCs but only at 100 µM, with limited membrane permeabilization being observed at their CC_50_. API and ENN B were also found to be able to cause membrane damage, but to a lesser extent. Finally, according to their effect on EGC viability, all regulated and non-regulated mycotoxins toxic to EGCs were found to be able to activate caspase-3/7, in accordance with their known ability to induce apoptosis in other cell types [14,30,31,32,33].

**Figure 10:**
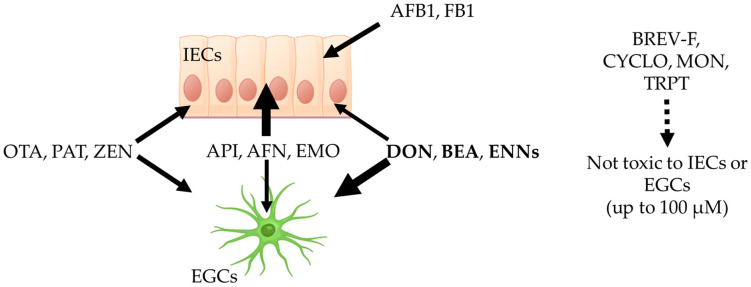
Comparison of the sensitivity of intestinal epithelial cells (IECs) and enteric glial cells (EGCs) to regulated and non-regulated mycotoxins on intestinal epithelial cells and EGCs. Brevianamide-F (BRV-F), cyclo-(L-Pro-L-Tyr) (CYCLO), moniliformin (MON), and tryptophol (TRPT) are not toxic to IECs or EGCs up to 100 µM. Aflatoxin B1 (AFB1) and fumonisin B1 (FB1) are only toxic to IECs and not toxic to EGCs up to 100 µM. Apicidin (API), aurofusarin (AFN), and emodin (EMO) are more toxic to IECs than to EGCs. Ochratoxin A (OTA), patulin (PAT), and zearalenone (ZEN) are equally toxic to IECs and EGCs. Deoxynivalenol (DON), beauvericin (BEA), and enniatins (ENNs) are more toxic to EGCs than to IECs. The image was generated using Biorender Premium.

The concentrations at which mycotoxins harm intestinal epithelial cells (IECs) are well characterized and described in the literature, allowing comparison with the toxic concentrations measured on EGCs in the present study to determine their selectivity (Figure 10). Brevianamide-F (BRV-F), cyclo-(L-Pro-L-Tyr) (CYCLO), moniliformin (MON), and tryptophol (TRPT) have been reported to be non-toxic to IECs [5] and were also found to be non-toxic to EGCs in the present work, suggesting they could have other cell/organ targets. Fumonisin B1 (FB1) [33] and aflatoxin B1 (AFB1) [34] cause toxicity to IECs at 21.0 and 39.1 µM, respectively, but were found not to be toxic to EGCs, demonstrating that these two mycotoxins selectively harm IECs. Aurofusarin (AFN) and emodin (EMO) have been shown to cause toxicity to IECs at 1.5, 19.1, and 19.0 µM, respectively [5]. Our data showed toxic concentrations for EGCs of 1.63–59.59, 79.51—> 100, and 40.01—> 100 µM for API, AFN, and EMO, respectively, demonstrating that these mycotoxins, although toxic to EGCs, preferentially harm IECs at lower concentrations. OTA [30,35], PAT [32], and ZEN [36] have been reported to affect IECs at 21.25, 14.43, and 62.1 µM, similar to the values obtained here on EGCs (i.e., 13.79–23.88, 9.19–38.02, and >100–31.75 µM for IC_50_ and CC_50_, respectively), showing that these toxins display similar toxicity against IECs and EGCs. Finally, deoxynivalenol (DON) [37], beauvericin (BEA) [29], and enniatins (ENNs) [29,31] have been reported to have toxic concentrations on IECs of 3.7, 3.9, and 1.1–4.6 µM, respectively, higher than the values found here on EGCs (i.e., as low as 0.19, 1.41, and 0.72 µM for DON, BEA, and ENNs, respectively), demonstrating that these mycotoxins are more toxic to EGCs than to IECs. This result suggests that, at least for these toxins, toxicity to EGCs may play an important role in their ability to affect the gut physiology, with alterations of EGCs occurring at lower exposure levels and/or prior to damage to the IECs.

Finally, in order to evaluate the risk, we compared the concentrations causing toxicity to EGCs to the mycotoxin concentrations susceptible to being achieved in real situations in humans. For that, intestinal and blood concentrations of toxins affecting EGCs were calculated based on their NOAEL, BMDL_10_, or LOAEL when these values are known, i.e., for BEA, DON, ENNs, OTA, PAT, and ZEN [38,39]. Calculation was not possible in the case of AFN, API, and EMO since no NOAEL, BMDL_10_, or LOAEL values are available for those toxins. From these concentrations in food, it was assumed that the mycotoxins are ingested in one meal, diluted in 1 L of gastrointestinal fluid, and are totally bioaccessible, as previously described [40]. The corresponding concentrations in µg/kg were converted to micromolar as a plausible dose in contact with intestinal cells in the gut lumen (Table 2). The amount reaching blood circulation was also determined, considering the absorption of each mycotoxin. The circulating concentration was estimated assuming a volume of 5 L of blood (Table 2). These gut and blood concentrations were compared to the toxic concentrations found on EGCs. From that comparison, it appears that of the eighteen mycotoxins tested, only a few may be at risk for EGCs in real situations, i.e., DON, ENNs, BEA, and PAT.

It will be important in future works to evaluate the impact of mycotoxins on EGC functions related to intestinal homeostasis [22,23,24,25,26,27]. Indeed, DON and OTA have been shown to alter astrocyte functions at sub-toxic doses [41,42]. Based on the fact that astrocytes and EGCs share many common features in terms of sensitivity to drugs and physiological functions [26], it will be worthwhile to evaluate if sublethal doses of mycotoxins affect EGC functions as observed with phycotoxins [43].

## 4. Conclusions

In conclusion, our data confirmed and extended the data of Gonkowski et al. [21] showing alteration of ENS caused by food-associated mycotoxins. Our data, generated using rat EGCs in vitro, confirmed the pioneering work of Rissato et al. [28], which showed that DON is able to affect the proliferation and viability of EGCs in vivo in rats. In addition, our in vitro data demonstrated that eleven other regulated or non-regulated (including emerging ones) mycotoxins (i.e., API, AFN, EMO, ENNs (ENN A, A1, B, B1), BEA, OTA, PAT, and ZEN) are able to affect the proliferation and/or viability of EGCs. API, DON, and cyclohexadepsipetides (i.e., ENNs and BEA) mycotoxins are the more worrying toxins with toxic concentrations as low as 190 nM. This study demonstrated that EGCs are the target of at least some food-associated mycotoxins, with their toxic effect being related to membrane damage, ROS production, and/or induction of apoptosis. Due to their pivotal role in the gut physiology [22,23,24,25,26,27], the toxic effect of mycotoxins on EGCs could lead to alterations of both ENS and IECs homeostasis and functions, further reinforcing the hypothesis, first enounced in 2010, of a link between mycotoxins and inflammatory bowel diseases (IBDs) [11]. Future works, including the evaluation of the effects of sub-toxic doses of mycotoxins on key functions of EGCs related to gut homeostasis, are required to confirm our hypothesis. Special attention should be given to the mycotoxins with toxic effects to EGCs at the lowest levels (i.e., DON, API, ENNs, and BEA), as such effects may happen at realistic dietary exposure.

## 5. Materials and Methods

### 5.1. Mycotoxins

Mycotoxins used in this study were selected from a global mycotoxin survey where more than 1100 feed samples from 44 countries were analyzed using a multi-analyte LC-MS/MS method. They were chosen based on their occurrence, their known or suspected toxicity to humans and/or animals, and their commercial availability [5]. Name, abbreviation, solvent used for preparation of the stock solutions, purity, and suppliers are indicated in Table 3. Stock solutions were stored at −20 °C. Working dilutions were freshly prepared in cell culture medium for each experiment.

### 5.2. Cell Culture, Antiproliferative, and Cytotoxicity Assays

Rat EGCs (ATCC^®^ CRL 2690™) obtained from ATCC (LGC Standards Molsheim, Molsheim, France) were maintained in complete culture medium corresponding to Dulbecco’s Modified Essential Medium (DMEM) supplemented with 10% fetal bovine serum (FBS), 1% L-glutamine, and 1% antibiotics (penicillin and streptomycin) (ThermoFisher, Illkirch-Graffenstaden, France). Cells were cultured in 75 cm^2^ flasks in a 5% CO_2_ incubator at 37 °C and passaged when reaching 90–95% confluence. Cell passage numbers 5 to 15 were used in all tests. Antiproliferative and cytotoxic assays were performed as previously described [44]. Briefly, cells were detached using a trypsin–EDTA solution (Thermofisher, Illkirch-Graffenstaden, France) and counted using a Malassez chamber. Cells were then diluted in complete culture media and seeded into 96-well cell culture plates (Greiner bio-one, Paris, France) at the appropriate cell density depending on the assay, i.e., approximately 5000 cells per well and 50,000 cells per well for antiproliferative and cytotoxic assays, respectively. Cells were then incubated for 12 h at 37 °C in a 5% CO_2_ incubator. The medium was removed, and the cells were incubated with increasing concentrations of mycotoxins diluted in complete culture medium (0 to 100 µM, ½ dilution). Negative controls were incubated with vehicle alone, i.e., ethanol (maximal volume of 10%) or DMSO (maximal volume of 1%). Cells were incubated for 48 h before the cell viability was measured using resazurin (toxicity assay kit from Sigma-Aldrich, Lyon, France) following the manufacturer’s instructions. After 4 h incubation at 37 °C, fluorescence intensity (excitation wavelength of 530 nm/emission wavelength of 590 nm) was measured using a microplate reader (Biotek, Synergy Mx, Colmar, France). The fluorescence values were normalized by the negative controls (vehicle-treated cells) and expressed as a percentage of proliferation or viability. The IC_50_ on cell proliferation (i.e., the concentration of mycotoxins reducing by 50% of the cell division) and the CC_50_ values on cell viability (i.e., the concentration of mycotoxins reducing the cell viability by 50%) were calculated using GraphPad^®^ Prism 10 software (San Diego, CA, USA) using nonlinear regression fitting.

### 5.3. Intracellular ROS Assay

Cellular oxidative stress was measured using the DCFDA assay as previously described [45]. Briefly, confluent EGCs seeded onto black fluorescent 96-well plates (Greiner bio-one, Paris, France) were loaded with the cell-permeable probe DCFDA diluted at a 30 µM final concentration in culture media. Cells were incubated in the dark at 37 °C for 30 min, allowing cellular esterases to deacetylate the compound into a non-cell-permeable and non-fluorescent compound. This intermediate is sensitive to ROS, which then oxidizes it into the fluorescent compound 2′,7′-dichlorofluorescein (DCF). After the 30 min incubation period, wells were aspirated, washed once with media, and refilled with new complete culture media. EGCs were then exposed to concentrations of mycotoxins corresponding to their CC_50_ on cell viability or to 100 µM. DMSO or ethanol was used as a negative control (i.e., untreated cells), with H_2_O_2_ at 500 µM final concentration being used as a positive control for the induction of intracellular ROS production. Fluorescence was measured after 1, 3, 6, 12, 24, or 48 h of incubation at 37 °C in the dark using a microplate reader (Biotek, Synergy Mx, Colmar, France) (excitation at 485 nm/emission at 535 nm). Data were analyzed and plotted using GraphPad^®^ Prism 10 software (San Diego, CA, USA).

### 5.4. Propidium Iodide Assay

Membrane integrity was measured using the propidium iodide (PI) assay as previously described [46]. Briefly, confluent EGCs seeded onto black fluorescent 96-well plates (Greiner bio-one, Paris, France) were then exposed to mycotoxins diluted into complete culture media containing 30 µM of PI. Tested concentrations corresponded to the CC_50_ of mycotoxins on cell viability or to 100 µM. DMSO or ethanol was used as a negative control (i.e., untreated cells), with Triton X-100 at 0.1% final concentration being used as a positive control for membrane permeabilization. Fluorescence was measured after 1, 3, 6, 12, 24, or 48 h of incubation at 37 °C in the dark using a microplate reader (Biotek, Synergy Mx, Colmar, France) (excitation at 530 nm/emission at 590 nm). Data were analyzed and plotted using GraphPad^®^ Prism 10 software (San Diego, CA, USA).

### 5.5. Caspase-3/7 Assay

The activation of caspase-3/7 was measured as an indicator of the induction of apoptosis using the CellEvent™ Caspase-3/7 Green Detection assay kit (Thermofisher, Illkirch-Graffenstaden, France) [47]. This assay is based on the use of a cell-permeable 4-amino acid peptide (DEVD) conjugated to a nucleic acid binding dye. In case of induction of apoptosis, activated caspase-3/7 cleaves the caspase-3/7 recognition sequence encoded in the DEVD, leading to the release of the nucleic acid binding dye and a bright fluorogenic signal. Briefly, confluent EGCs seeded onto black fluorescent 96-well plates (Greiner bio-one, Paris, France) were then exposed to mycotoxins diluted into complete culture media containing DEVD conjugated peptide diluted according to the manufacturer’s instructions. Tested concentrations corresponded to the CC_50_ of mycotoxins on cell viability or to 100 µM. DMSO or ethanol was used as a negative control (i.e., untreated cells), and staurosporine at a 10 µM final concentration was used as a positive control for caspase-3/7 activation. Fluorescence was measured after 1, 3, 6, 12, 24, or 48 h of incubation at 37 °C in the dark using a microplate reader (Biotek, Synergy Mx, Colmar, France) (excitation at 502 nm/emission at 530 nm). Data were analyzed and plotted using GraphPad^®^ Prism 10 software (San Diego, CA, USA).

### 5.6. Statistical Analysis

All experiments were conducted in independent triplicates (*n* = 3). Two-way ANOVA analyses and t-tests were used to address significant differences between mean values with significance set at *p* < 0.05 (GraphPad^®^ Prism 10 software).

## Figures and Tables

**Table 1:** Impact of mycotoxins on proliferation and viability of EGCs. EGCs were exposed for 48 h to increasing concentrations of mycotoxins before determination of their 50% inhibitory effect on proliferation (IC_50_) or cell viability (CC_50_). IC_50_ and CC_50_ values were determined from Figure 1 and Figure 2 and are expressed as means ± S.D. (in µM).

	AFB1	DON	FB1	OTA	PAT	ZEN
IC_50_ on proliferation	>100	0.19 ± 0.07	>100	13.79 ± 1.13	9.19 ± 0.96	>100
CC_50_ on viability	>100	5.06 ± 0.48	>100	23.88 ± 1.36	38.02 ± 11.37	31.75 ± 4.94
	BEA	ENN A	ENN A1	ENN B	ENN B1	CYCLO
IC_50_ on proliferation	1.41 ± 0.20	0.93 ± 0.13	1.08 ± 0.34	1.40 ± 0.18	0.92 ± 1.07	>100
CC_50_ on viability	1.91 ± 0.45	1.05 ± 0.11	0.86 ± 0.09	0.72 ± 0.16	2.14 ± 0.17	>100
	AFN	API	BRV-F	EMO	MON	TRPT
IC_50_ on proliferation	79.51 ± 3.68	1.63 ± 0.21	>100	40.01 ± 2.70	>100	>100
CC_50_ on viability	>100	59.59 ± 10.27	>100	>100	>100	>100

**Table 2:** Estimation of the intestinal and blood concentrations of mycotoxins found to be toxic to EGCs. Intestinal and blood concentrations of mycotoxins found to be toxic to EGCs were calculated based on their BMDL/NOAEL/LOAEL levels. The calculated gut and blood concentrations were compared to the concentrations causing antiproliferative (IC_50_) or cytotoxic (CC_50_) effects to EGCs. Values in bold are below calculated gut and/or blood concentrations, suggesting potential risk.

Mycotoxin	Toxicological Threshold	Value µg/kg bw/Day	Gastrointestinal Concentration at the BMDL/NOAEL Level (µM) *	Blood Concentration at the BMDL/NOAEL Level (µM) **	Antiproliferative (IC_50_)/Cytotoxic (CC_50_) Concentrations on EGCs (µM)
DON	NOAEL	100	23.6	3.31	0.19/5.06
OTA	*Non-neoplastic effects* BMDL_10_	14.5	2.5	0.43	13.79/23.88
PAT	NOAEL	40	19.5	0.08	9.19/38.02
ZEN	NOAEL	100	22	3.5	>100/31.75
ENNs, BEA	LOAEL ***	17,000	18	2.9	0.92/0.72

* The gastrointestinal concentration of mycotoxins was calculated considering a body weight of 70 kg and a dilution of the toxin in 1 L of gastrointestinal fluid. ** The blood concentration of mycotoxins was calculated considering a total blood volume of 5 L and taking into account the specific absorption of each toxin (95% for AFB1, 85% for OTA, 80% for ZEN, 70% for DON, 4% for FB1, 2% for OTA, and 80% for ENNs). *** For ENNs and BEA, the LOAEL was determined based on the LOAEL for fusafungine (an antibiotic and anti-inflammatory nasal/oromucosal spray agent containing a mixture of ENNs).

**Table 3:** Data regarding the mycotoxins used in this study.

Name	Abbreviation	Solvent Used	Purity	Supplier
Aflatoxin B1	AFB1	DMSO	>98%	Sigma Aldrich, Lyon, France
Apicidin	API	DMSO	>98%	Sigma Aldrich
Aurofusarin	AFN	DMSO	>97%	Santa Cruz Biotechnology, Dallas, TX, USA
Beauvericin	BEA	Ethanol	>97%	Sigma Aldrich
Brevianamide-F	BRV-F	Ethanol	>95%	BioAustralis, Smithfield, Australia
Cyclo-(L-Pro-L-Tyr)	CYCLO	Ethanol	>98%	BioAustralis
Deoxynivalenol	DON	Ethanol	>98%	Sigma Aldrich
Emodin	EMO	DMSO	>90%	Sigma Aldrich
Enniatins	ENNs	Ethanol	>99%	Sigma Aldrich
Fumonisin B1	FB1	DMSO	>98%	Sigma Aldrich
Moniliformin	MON	Ethanol	>95%	Sigma Aldrich
Ochratoxin A	OTA	Ethanol	>95%	Sigma Aldrich
Patulin	PAT	Ethanol	>98%	Sigma Aldrich
Tryptophol	TRPT	DMSO	>97%	Sigma Aldrich
Zearalenone	ZEN	DMSO	>98%	Sigma Aldrich

## Data Availability

The authors declare that the data supporting the findings of this study are available within the paper. Should any raw data files be needed in another format, they are available from the corresponding author upon reasonable request.
